# Entropy Analysis of Implicit Heat Fluxes in Multi-Temperature Mixtures

**DOI:** 10.3390/e26090723

**Published:** 2024-08-24

**Authors:** A. D. Kirwan, Mehrdad Massoudi

**Affiliations:** 1 School of Marine Science and Policy, University of Delaware, Robinson Hall, Newark, DE 19716, USA; 2 U.S. Department of Energy, National Energy Technology Laboratory (NETL), 626 Cochrans Mill Road, Pittsburg, PA 15236, USA; mehrdad.massoudi@netl.doe.gov

**Keywords:** mixtures, entropy, two temperatures, non-Fourier conduction, Maxwell–Cattaneo model, constitutive modeling

## Abstract

We propose new implicit constitutive relations for the heat fluxes of a two-temperature mixture of fluids. These relations are frame-indifferent forms. However, classical explicit forms of the stress tensors and the interaction forces (specified as explicit forms of constitutive relations) as given in mixture theory are used. The focus here is to establish constraints imposed on the implicit terms in the heat fluxes due to the Second Law of Thermodynamics. Our analysis establishes that the magnitude of the explicit entropy production is equal to or greater than that of the implicit entropy production.

## 1. Introduction

Mathematically, the purpose of constitutive relations is to connect kinematical, mechanical, chemical, electro-magnetic, and thermal fields with the governing balance equations and, as a whole, to provide a theory that is solvable for properly posed problems. Deriving constitutive relations for the stress tensors, the heat fluxes, and the interaction forces are among the outstanding issues of research in multi-component flows. For example, Clayton [1] used mixture theory to study the interactions between a deformable solid and a compressible fluid phase, while allowing distinct temperatures, entropy, and internal energy fields. An important and often neglected issue with the application of mixture theory is the specification of boundary conditions. In a recent study, Klika and Votinská [2] discussed the significance of boundary conditions which are needed in mixture theory. They suggested using the Maximum Entropy principle to obtain estimates for the state variables needed in specifying the boundary conditions.

Theories dealing with a mixture of two components, for example, two fluids, with two different temperatures usually assume that the constitutive parameters for the viscous stress tensors $T_{1},T_{2}$ and heat fluxes $q_{1},q_{2}$ depend on the densities $\rho_{1},\rho_{2}$, the temperatures $\theta_{1},\theta_{2}$, the velocities $v_{1},v_{2}$, and possibly their time derivatives and gradients. In both of the temperature theories of mixtures that we are aware of, the form of $q_{1}$ and $q_{2}$ is based on Fourier’s law of heat conduction, a well-known explicit constitutive equation. In most of these theories, it is also assumed that at some time, the two temperatures reach an equilibrium, and thus there is usually an expression for the heat flux vector of the mixture $q_{m}$. However, in [3], we argue that in reality, there are many processes that can delay the approach to such an equilibrium. For such cases, implicit forms of $q_{1}$ and $q_{2}$ may be more appropriate since they introduce the concept of time (the relaxation or response time) into the constitutive relations. Here, we extend the ideas developed in [4] to the case where the two qs include implicit forms.

Giovine [5] developed a scheme for an immiscible mixture considering microstructural effects such as diffusion and adsorption phenomena, chemical reactions, etc., while using the internal constraints in entropy inequality based on extended thermodynamics. Gorgone et al. [6] presented a thermodynamical analysis for a binary mixture of Korteweg fluids with two velocities and two temperatures, where the constitutive functions were allowed to depend on the diffusion velocity and the specific internal energies of both constituents, among other variables. Málek and Souček [7] used the theory of interacting continua, i.e., mixture theory, to study the mixture of two fluids with heat conduction with different temperatures.

Traditionally, constitutive equations for the fluxes of heat and momentum are explicit statements. That is, they are expressed explicitly in terms of polynomials of appropriate objective and potentially observable quantities. Fourier’s law for the heat flux and the Navier–Stokes formulation for the stress tensor of fluids are two hugely successful examples of this approach. Entropy analysis of such explicit relations has played an important role in establishing constraints on the consequent phenomenological coefficients. With explicit formulations, the entropy flux is connected to the energy equation in a consistent fashion through the free energy, thus providing a crucial link between the heat flux and the entropy flux. The essence of this approach is to express entropy production as a sum of products of “forces and fluxes” [8] and then apply the non-negativity of the entropy production terms to obtain coefficient restrictions that can be tested both theoretically and experimentally. Eckart [9,10,11], Onsager [12,13], and Kondepudi and Prigogine [14] provided classic examples of this approach.

Wang and Hutter [15] provided a thorough review of the irreversible thermodynamics from a phenomenological point of view, where the significance of entropy constraints was discussed (see also Bejan [16]). Liu [17] reviewed three distinct scientific areas, namely, equilibrium and non-equilibrium thermodynamics, statistical mechanics, and quantum mechanics. These theories seem to be disconnected from each other since they use different principles based on different ideas as developed in these different fields. More recently, Kirwan [4] used this approach for an entropy analysis of a mixture with different component temperatures, and Massoudi and Kirwan [18] used it for a heat conducting suspension.

An important development in continuum physics is the introduction of an implicit constitutive equation for the heat flux vector and the stress tensor. Morgan [19] and Raj [20,21] provided the theoretical foundation for this approach. Most implicit constitutive models specify the heat flux as a rate equation, i.e., the Maxwell–Cattaneo (MC) equation. This has three important advantages over the Fourier’s Law model: the heat flux propagates at a finite speed, the equation can be made frame indifferent, i.e., objective, and there is a second degree of freedom, i.e., the relaxation time, which can be used in a comparison of the theory with observations. See Christov [22] and Massoudi and Mehrabadi 2010 [23] for examples.

In principle, the MC equation can be solved to obtain an expression for the heat flux. Unfortunately, implicit constitutive models typically require solutions to frame-invariant rate equations, where analytic solutions are available only for a few limiting cases. This severely limits any general entropy analysis to extremely simple situations. The lack of a general approach raises fundamental questions pertinent to an entropy analysis for mixtures: What if the constitutive equations are a combination of explicit and implicit equations? How can the entropy flux be connected with an implicit equation for the heat flux? Are there second law restrictions on relaxation times? Can an implicit heat flux paradigm be extended to mixtures?

To our knowledge, entropy production for implicit heat flux constitutive relations in mixtures with different temperatures has not yet been studied. Our approach differs somewhat from traditional entropy analyses of mixtures in that we rely on the Green Adkins Massoudi (GAM) limiting case principle [4]: *in the absence of one component, the governing equations and the constitutive relations must reduce to the appropriate forms for a single component.* This principle is a modification of Truesdell’s [24] and Massoudi’s [25] third metaphysical model.

The next section summarizes the essential theoretical background of mixture theory. Section 3 very briefly reviews recent developments in explicit and implicit models of heat flux in single component materials, specifically, the Maxwell–Cattaneo model. Section 4 proposes an extension of this model for a mixture of two fluids with different temperatures. Here, we establish the central result of the analysis: entropy production by explicit terms in the constitutive equations is no greater than that of the implicit terms. The paper concludes with a statement on some broader implications of this result.

## 2. Theoretical Background

The mass, momentum, and energy conservation equations for each mixture component are well known [24,26] to be

(1) $$\begin{array}{ccc} \; & \; & D_{\alpha }\rho_{\alpha }+\rho_{\alpha }\nabla \cdot v_{\alpha }={\hat{c}}_{\alpha }\\ \; & \; & \rho_{\alpha }D_{\alpha }v_{\alpha }=\nabla \cdot \Sigma_{\alpha }+\rho_{\alpha }b_{\alpha }+{\hat{m}}_{\alpha }\\ \; & \; & \Sigma_{\alpha }-\Sigma_{\alpha }^{T}={\hat{M}}_{\alpha }\\ \; & \; & \rho_{\alpha }D_{\alpha }\epsilon_{\alpha }=\Sigma_{\alpha }:\nabla v_{\alpha }-\nabla \cdot q_{\alpha }+\rho_{\alpha }r_{\alpha }^{\epsilon }+{\hat{\epsilon }}_{\alpha }. \end{array}$$

Here, ∇ is the gradient operator, $D_{\alpha }\left(\right)$ is the material derivative following constituent α, i.e., in general for any scalar β, $D_{\alpha }\beta =\partial \beta /\partial t+v_{\alpha }\cdot \nabla \beta ,\alpha =1,2$, and for any vector z$D_{\alpha }z=\partial z/\partial t+v_{\alpha }\cdot \nabla z$. The remaining terms $\rho_{\alpha },v_{\alpha },\epsilon_{\alpha }$ are, respectively, the density, velocity, and internal energy density of that component. On the right-hand side of (1), $\Sigma_{\alpha },b_{\alpha },q_{\alpha }$, and $r_{\alpha }^{\epsilon }$ are, respectively, the stress tensor, externally applied body force, heat flux vector, and the exterior energy supply to constituent α. The superscript *T* is the transpose operation. Finally, $\hat{c},{\hat{m}}_{\alpha },{\hat{M}}_{\alpha }$, and ${\hat{\epsilon }}_{\alpha }$ account for the appropriate interactions of mass, momentum, torque, and energy between the α components.

Dynamical considerations stipulate that the interaction terms on the RHS of (1) satisfy

(2) $$\sum_{\alpha }{\hat{c}}_{\alpha }=\sum_{\alpha }\left({\hat{m}}_{\alpha }+{\hat{c}}_{\alpha }v_{\alpha }\right)=\sum_{\alpha }{\hat{M}}_{\alpha }=\sum_{\alpha }\left[{\hat{\epsilon }}_{\alpha }+{\hat{m}}_{\alpha }\cdot v_{\alpha }+{\hat{c}}_{\alpha }\left(\epsilon_{\alpha }+\kappa_{\alpha }\right)\right]=0.$$

In this last equation, $\kappa_{\alpha }$ is the kinetic energy of the α constituent. Note, however, when (1) is summed over all constituents, the resulting equations on the LHS are not quite the same as those of a single body. One reason is that the $\sum_{\alpha }D_{\alpha }$ terms introduce “diffusion velocities”, which have no counterpart in the equations for a single body [27]. As noted in [4] and discussed below, dynamic constraints imposed on the constitutive equations by the summation of the RHS terms may conflict with the Second Law. In a related study, Bertei et al. [28] used methods of non-equilibrium thermodynamics to show the significance of the chemical potential gradient at a constant temperature as the driving force for mass diffusion, allowing for the implicit thermo-diffusion effect to be absent and where the Dufour effect is negligibly small.

A general solution of (1) requires constitutive equations for ${\hat{c}}_{\alpha },{\hat{m}}_{\alpha },\Sigma_{\alpha },\hat{M},q_{\alpha },{\hat{\epsilon }}_{\alpha }$ and $r_{\alpha }^{\epsilon }$. For simplicity in the subsequent analysis, we assume no chemical interactions between the constituents; thus, ${\hat{c}}_{\alpha }=0$. Our focus is on the impact that an implicit formulation of the heat fluxes $q_{\alpha }$ has on a Second Law analysis for the mixture. Explicit constitutive equations for $\Sigma_{\alpha },{\hat{m}}_{\alpha }$ and $q_{\alpha }$ are given in [4] as

(3) $$\begin{array}{ccc} \; & \; & \Sigma_{\alpha }=\phi_{\alpha }\left[\lambda_{\alpha \alpha }d_{\alpha }I+2\mu_{\alpha \alpha }d_{\alpha }+\phi_{\beta }\left(\lambda_{\alpha \beta }d_{\beta }I+2\mu_{\alpha \beta }d_{\beta }\right)+\phi_{\beta }\nu_{\alpha \beta }\left(w_{\alpha }-w_{\beta }\right)\right]\\ \; & \; & \hat{m_{\alpha }}=-\phi_{\alpha }\phi_{\beta }\left(l_{\alpha \beta }u_{\alpha \beta }+\sum_{\gamma }p_{\alpha \beta \gamma }\nabla \theta_{\alpha }\right)\\ \; & \; & q_{\alpha }=-\phi_{\alpha }\left[k_{\alpha \alpha }\nabla \theta_{\alpha }+\phi_{\beta }\left(k_{\alpha \beta }\nabla \theta_{\beta }+h_{\alpha \beta }u_{\alpha \beta }\right)\right] \end{array}$$

for α≠β. In the first equation, $d_{\gamma },d_{\gamma }$ and $w_{\gamma }$, respectively, are the divergence, deviator, and spin components of the velocity gradient of the γ component of the mixture, and $u_{\alpha \beta }=v_{\alpha }-v_{\beta }$ is the velocity difference, an objective vector. Also, I is the identity tensor and λ,μ, and ν are phenomenological coefficients. Finally, $\phi_{\alpha }$ is the volume fraction of the α constituent, defined as $\phi_{\alpha }=V_{m}/{\hat{V}}_{m}$, where $V_{m}$ is the volume of substance α in the mixture and ${\hat{V}}_{m}$ is the volume occupied by α if there were no mixture. For additional simplicity, we assume the mixture is saturated and there are no volume voids, i.e., $\sum_{\alpha }\phi_{\alpha }=1$. This is the same weight function used by [4,25] to ensure that the constitutive equations satisfy the GAM limiting case principle, i.e., they reduce to the appropriate form when the volume fraction of a constituent vanishes.

As noted above, (2) imposes constraints on the phenomenological coefficients in (3). These are

(4) $$\begin{array}{ccc} \; & \; & \nu_{\alpha \beta }=\nu_{\beta \alpha }\\ \; & \; & l_{\alpha \beta }=l_{\beta \alpha }\\ \; & \; & p_{\alpha \beta \gamma }=p_{\beta \alpha \gamma }. \end{array}$$

However, that reference also states that these constraints may be inconsistent with the Second Law for multi-temperature mixtures. Below, constraints imposed by the Second Law are developed, which reduce to (4) when the constituent temperatures are the same.

## 3. Constitutive Modeling of the Heat Flux Vector

Before considering the constitutive modeling of the heat flux vectors in mixtures with different temperatures, it is appropriate to review some basic concepts for the heat flux vector for a single component substance.

The classical theory of heat conduction, first proposed by Fourier and later generalized by Duhamel (see [24] for an interesting history on this topic), assumes that the constitutive relation for the heating flux h for a (solid-like) body is a linear function of the temperature gradient

(5) h=K(θ,F)·∇θ.

Here, F is the deformation gradient, and K is the thermal conductivity tensor. According to Winterton [29], Fourier first stated that heat conduction depends on the temperature gradient and not on the temperature difference between the two adjacent parts of a solid body. For an isotropic material, this equation reduces to the classical Fourier’s law of heat conduction

(6) q=−k∇θ

where *k* is generally assumed to be constant. This equation is an example of an explicit scheme, where the unknown, q, is directly, i.e., explicitly, related to the measurable variable, the gradient of temperature ∇θ. There have been many attempts to generalize Fourier’s heat conduction law. For example, the thermal conductivity is assumed to depend on the volume fraction or the shear rate, etc. This is very similar to attempts made in rheology where power-law models for viscosity are assumed to depend on volume fraction or shear rate. And just as in rheology, in order to capture rate-dependent effects, models such as the Oldroyd or integral models have been developed. As noted earlier, there have been many comparable attempts to develop a rate-type heat flux vector for various materials.

In fact, as early as 1867, Maxwell recognized that if the classical Fourier’s law of heat conduction is used in the energy equation, one obtains a parabolic transport equation (diffusion type). This means that when the material is subjected to a thermal disturbance, the effects are felt instantaneously everywhere, i.e., the thermal signal propagates with infinite speed. That is, in the absence of internal heating, and for a homogenous isotropic material, one obtains the parabolic heat transport equation:

(7) $$k\nabla^{2}\theta =\rho c\frac{\partial \theta }{\partial t}.$$

The issue of an infinite propagation speed can be addressed by considering the heat flux vector as an implicit function of ∇θ in the form:

(8) f(q,∇θ,θ)=0.

An early example of such a model is due to Fox [30], who proposed a rate-type constitutive equation for the heat flux vector q that could be used by imposing restrictions due to frame indifference, material symmetry, and entropy inequality. He obtained the following equation

(9) τDq+q=−k∇θ.

Here, Dq=Dq−W·q is the objective Jaumann derivative of q, and $W=(\nabla v-{\left(\nabla v\right)}^{T})/2$ is the spin tensor of the flow field. There are two phenomenological parameters in this model: τ, a positive relaxation time, and k, the thermal conductivity. Equation (9) is often referred to as the Maxwell–Cattaneo (MC) equation. It reduces to the classic Fourier’s law of heat conduction when either τ=0 or Dq=0.

If (9) is substituted in the energy equation, one obtains a hyperbolic-type heat transport equation $k\nabla^{2}\theta =\rho c\left(\partial \theta /\partial t+\partial^{2}\theta /\partial t^{2}\right)$. This predicts a finite speed, $V_{T}={\left(k/\rho c\tau \right)}^{1/2}$ for heat propagation. Chandrasekariah [31] and Chester [32] studied second sound in elastic bodies and reported that the relaxation time can be estimated as $\tau =3k/(S^{2}C)$, where *S* is a sound velocity and *C* is a specific heat of the solid. These results show that the quandary of the infinite propagation speed of heat pulses is eliminated with the CV equation.

It is worth noting that the issue of an infinite propagation speed is a long-standing issue in other disciplines. Massoudi and Mehrabadi [23,33] used the Fox model [30] to study implicit heat flux vectors in thermoelasticity and a porous-like granular material. A hyperbolic equation similar to (9) was first used in telegraphy for detecting breaks in telegraphic cables; consequently, it is often referred to as the “telegraph equation”. Kirwan and Kump [34] found that this equation surpassed a Fickian diffusion model of the migration of geochemical species in the environment.

Of course, other types of frame-indifferent derivatives can also be used. For example, Christov [22] derived a frame-indifferent form of the MC model by using an Oldroyd-type time derivative of the type:

(10) $$Dq=\left[\frac{Dq}{Dt}-q\cdot \nabla v+\left(\nabla \cdot v\right)q\right].$$

The next section develops an implicit model for the two heat fluxes for a two-temperature mixture of two fluids and obtains constraints on these models imposed by the Second Law.

## 4. Entropy Analysis

The remaining portion of this report deals with a two-component mixture with distinct temperatures for each component. Presumably, each α component has an entropy balance equation of the general form

(11) $$\rho_{\alpha }D_{\alpha }\eta_{\alpha }+\nabla \cdot j_{\alpha }-\rho r_{\alpha }^{\eta }={\hat{\eta }}_{\alpha }.$$

Here, $\eta_{\alpha }$ is the entropy of the α constituent, $r_{\alpha }^{\eta }$ is the external supply of entropy, $j_{\alpha }$ is the entropy flux, and ${\hat{\eta }}_{\alpha }$ is the internal generation of entropy, all for the α constituent. The principle of non-negative generation of entropy for the mixture demands that

(12) $$\sum_{\alpha }{\hat{\eta }}_{\alpha }\geq 0.$$

For explicit constitutive equations, the choice

(13) $$\begin{array}{ccc} \; & \; & j_{\alpha }=q_{\alpha }/\theta_{\alpha }\\ \; & \; & r_{\alpha }^{\eta }=r_{\alpha }^{\epsilon }/\theta_{\alpha } \end{array}$$

leads to physically consistent results (see [4]). That analysis relies on multiplying the energy equation of (1) by $\theta_{\alpha }$, subtracting (11) to obtain

(14) $${\hat{\eta }}_{\alpha }=\left[\rho_{\alpha }\left(\theta_{\alpha }D_{\alpha }\eta_{\alpha }-D_{\alpha }\epsilon_{\alpha }\right)-\left(q_{\alpha }\cdot \nabla \theta_{\alpha }\right)/\theta_{\alpha }+M_{\alpha }:\nabla v_{\alpha }+{\hat{\epsilon }}_{\alpha }\right]/\theta_{\alpha }.$$

Assuming each mixture component has its own free energy $\psi_{\alpha }-\theta_{\alpha }\eta_{\alpha }$, then (14) reduces to

(15) $${\hat{\eta }}_{\alpha }=\left[M_{\alpha }:\nabla v_{\alpha }-{\hat{m}}_{\alpha }\cdot v_{\alpha }-\left(q_{\alpha }\cdot \nabla \theta_{\alpha }\right)/\theta_{\alpha }-\rho_{\alpha }\left(D_{\alpha }\psi_{\alpha }+\eta_{\alpha }D_{\alpha }\theta_{\alpha }\right)\right]/\theta_{\alpha }.$$

In arriving at (15), $\sum_{\alpha =1}^{2}\left({\hat{\epsilon }}_{\alpha }+{\hat{m}}_{\alpha }\cdot v_{\alpha }\right)=0$ was assumed.

In classical thermodynamic theory and many mixture theories with a common temperature, it is assumed that each constituent has equilibrium conditions specified by $\rho_{\alpha }\left(D^{\alpha }\psi_{\alpha }+\eta_{\alpha }D^{\alpha }\theta_{\alpha }\right)=0$. This assumption reduces the entropy production for each constituent to

(16) $${\hat{\eta }}_{\alpha }=\left[M_{\alpha }:\nabla v_{\alpha }-{\hat{m}}_{\alpha }\cdot v_{\alpha }-\left(q_{\alpha }\cdot \nabla \theta_{\alpha }\right)/\theta_{\alpha }\right]/\theta_{\alpha }\geq 0.$$

The three entropy-generating terms in (16) can be viewed as products of thermodynamic “forces” and “fluxes” [8]. Furthermore, they are divided into a tensor product, $M_{\alpha }:\nabla v_{\alpha }$, and two vector products, ${\hat{m}}_{\alpha }\cdot v_{\alpha }$ and $\left(q_{\alpha }\cdot \nabla \theta_{\alpha }\right)/\theta_{\alpha }$. The sum of the tensor and vector products are independent, and each sum must satisfy (16) separately [8]. That is,

(17) $$\begin{array}{ccc} \; & \; & C_{1}\;M_{1}:\nabla v_{1}+C_{2}\;M_{2}:\nabla v_{2}\geq 0\\ \; & \; & C_{1}\;{\hat{m}}_{1}\cdot v_{1}+C_{1}^{2}\;q_{1}\cdot \nabla \theta_{1}+C_{2}\;{\hat{m}}_{2}\cdot v_{2}+C_{2}^{2}\;q_{2}\cdot \nabla \theta_{1}\leq 0. \end{array}$$

Consistent with the previous analysis of multi-temperature mixtures [4], we now use the “coldness” $C_{\alpha }=\theta_{\alpha }^{-1}$ of each constituent rather than its temperature $\theta_{\alpha }$.

Entropy analysis that involves explicit constitutive equations establishes the signs and relative magnitudes of the phenomenological coefficients that appear in these equations. For mixtures with distinct constitutive properties, a modest extension of that approach is required to determine the positive/negative definiteness of quadratic forms. Following [4], we employ the method of principal minors. Here, the production terms of (17) are organized into systems of matrix products of the form $x\cdot P\cdot x^{T}$, where the elements of x are objective functions, and P are matrices, not necessarily symmetric, composed of phenomenological coefficients. The set of principal minors of P determines the positive/negative definiteness of $x\cdot P\cdot x^{T}$. The requirement that they all be positive/negative provides thermodynamic constraints on the signs and relative sizes of the coefficients. We apply this method here even though some of the constitutive equations are implicit.

Consider first the tensor entropy production given by the first equation of (17). The stress constitutive equations (3) are explicit, so they are amenable to the method of principal minors. They were analyzed in [4], who concluded

(18) $$\begin{array}{ccc} \; & \; & \phi_{1}\lambda_{11}C_{1},\;\phi_{2}\lambda_{22}C_{2}\geq 0\\ \; & \; & \lambda_{11}\lambda_{22}C_{1}C_{2}\geq \left(\phi_{1}\phi_{2}/4\right){\left(\lambda_{12}C_{1}+\lambda_{21}C_{2}\right)}^{2}\\ \; & \; & \phi_{1}\mu_{11}C_{1},\;\phi_{2}\mu_{22}C_{2}\geq 0\\ \; & \; & \mu_{11}\mu_{22}C_{1}C_{2}\geq \left(\phi_{1}\phi_{2}/4\right){\left(\mu_{12}C_{1}+\mu_{21}C_{2}\right)}^{2}\\ \; & \; & \nu_{12}C_{1}=\nu_{21}C_{2}\geq 0. \end{array}$$

When $C_{1}=C_{2}$, (18) reduces to the results reported by Atkin and Craine [35,36] for mixtures with uniform temperatures. Note, however, if the temperatures of the two constituents are different, it is not possible to satisfy the ${\hat{M}}_{1}+{\hat{M}}_{2}=0$ dynamic constraint imposed by (2), unless ${\hat{M}}_{1}={\hat{M}}_{2}=0$.

Now, it is necessary to generalize the MC constitutive heat flux vector model for a mixture of two constituents with distinct temperatures that lead to testable results from a Second Law analysis. To this end, we propose:

(19) $$\begin{array}{ccc} \; & \; & \phi_{1}\left(\tau_{11}D_{1}q_{1}+\phi_{2}\tau_{12}D_{2}q_{2}\right)+q_{1}=-\phi_{1}\left[k_{11}\nabla \theta_{1}+\phi_{2}\left(k_{12}\nabla \theta_{2}+h_{12}u_{12}\right)\right]\\ \; & \; & \phi_{2}\left(\tau_{22}D_{2}q_{2}+\phi_{1}\tau_{21}D_{1}q_{1}\right)+q_{2}=-\phi_{2}\left[k_{22}\nabla \theta_{2}+\phi_{1}\left(k_{21}\nabla \theta_{1}+h_{21}u_{21}\right)\right]. \end{array}$$

The subscript on $D_{\alpha }$ is the Jaumann derivative following the flow of that constituent, and $\tau_{\alpha \beta },k_{\alpha \beta }$, and $h_{\alpha \beta }$ are phenomenological parameters. These equations reduce to (9) when only one constituent is present and also reduce to the explicit form studied in [4] when either $\tau_{\alpha \beta }$ or $D_{\alpha }q_{\alpha }=0$. It is noteworthy that this expression contains both implicit and explicit terms. The implicit terms $D_{\alpha }q_{\alpha }$ are on the left-hand side, and the explicit terms on the right-hand side.

Using (19) in the second inequality of (17), we see that the vector entropy production terms are a combination of explicit and implicit entropy production terms. The objective vectors are $u_{ab},\nabla \theta_{\alpha }$, and $D_{\alpha }$, while the phenomenological parameters are $k_{\alpha \beta },h_{\alpha \beta },l_{\alpha \beta },\tau_{\alpha \beta }$, and $p_{\alpha \beta \gamma }$. The explicit terms in (17) were analyzed in [4], wherein it was shown that with the stipulations $l_{\alpha \beta }C_{\alpha }=l_{\beta \alpha }C_{\beta }=l$ and $p_{\alpha \beta \gamma }C_{\gamma }=-p_{\beta \alpha \gamma }C_{\gamma }=p$, they could be reduced to the quadratic form $\left[\nabla \theta_{1},\nabla \theta_{2},u_{\alpha \beta }\right]\cdot P\cdot {\left[\nabla \theta_{1},\nabla \theta_{2},u_{\alpha \beta }\right]}^{T}$, where

(20) $$P=\left[\begin{array}{ccc} \phi_{1}k_{11}C_{1}^{2} & \left(\phi_{1}\phi_{2}/2\right)\left(k_{12}C_{1}^{2}+k_{21}C_{2}^{2}\right) & \left(\phi_{1}\phi_{2}/2\right)\left(h_{12}C_{1}^{2}+p\right)\\ \left(\phi_{1}\phi_{2}/2\right)\left(k_{12}C_{1}^{2}+k_{21}C_{2}^{2}\right) & \phi_{2}k_{22}C_{2}^{2} & -\left(\phi_{1}\phi_{2}/2\right)\left(h_{12}C_{2}^{2}+p\right)\\ \left(\phi_{1}\phi_{2}/2\right)\left(k_{12}C_{1}^{2}+k_{21}C_{2}^{2}\right) & -\left(\phi_{1}\phi_{2}/2\right)\left(h_{12}C_{2}^{2}+p\right) & \phi_{1}\phi_{2}l \end{array}\right].$$

The Second Law requires P to be negative definite. Inequalities involving the phenomenological coefficients arising from this constraint are discussed in [4].

We write the explicit vector entropy production terms in (17) as

(21) $$C_{1}^{2}\phi_{1}\tau_{11}D_{1}q_{1}\cdot \nabla \Theta_{1}+C_{2}^{2}\phi_{2}\tau_{22}D_{2}q_{2}\cdot \nabla \Theta_{2}+\phi_{1}\phi_{2}\left(C_{1}^{2}\tau_{12}D_{2}q_{2}\cdot \nabla \theta_{1}+C_{2}^{2}\tau_{21}D_{1}q_{1}\cdot \nabla \theta_{2}\right)=E.$$

This equation is consistent with the general form for entropy production terms, in that it involves the product of two objective vectors, one of which contains phenomenological parameters. However, the signs of the objective vectors are problem dependent. For example, $D_{1}q_{1}\cdot \nabla \theta_{1}$ may be positive during one period of time and negative during another period. Moreover, it is not possible a priori to establish the relative magnitudes of, say, $D_{1}q_{1}\cdot \nabla \theta_{1}$ vs. $D_{1}q_{1}\cdot \nabla \theta_{2}$.

Using (19) reduces the vector entropy production terms to

(22) $$-E-\left[\nabla \theta_{1},\nabla \theta_{2},u_{\alpha \beta }\right]\cdot P\cdot {\left[\nabla \theta_{1},\nabla \theta_{2},u_{\alpha \beta }\right]}^{T}\geq 0.$$

As it was established in [4] that $\left[\nabla \theta_{1},\nabla \theta_{2},u_{\alpha \beta }\right]\cdot P\cdot {\left[\nabla \theta_{1},\nabla \theta_{2},u_{\alpha \beta }\right]}^{T}\leq 0$, we conclude that

(23) $$\left|E|\leq \right|\left[\nabla \theta_{1},\nabla \theta_{2},u_{\alpha \beta }\right]\cdot P\cdot {\left[\nabla \theta_{1},\nabla \theta_{2},u_{\alpha \beta }\right]}^{T}|$$

is the Second Law constraint for implicit constitutive equations. The essence of (22) and (23) is that the magnitudes of the explicit entropy production are equal or greater than that of implicit entropy production. Our fundamental conclusion, then, is that for entropy analyses involving explicit constitutive equations of the general form given by (19), the magnitude of the explicit entropy production is never less than the implicit entropy production.

## 5. Discussion

The analysis here focuses on entropy production for a mixture of two fluids with different temperatures and with implicit constitutive equations for the heat fluxes. The explicit production terms must be appropriately positive or negative definite. This constraint imposes strict sign conditions on the explicit phenomenological coefficients. However, the signs of the implicit production terms involve rate equations, which may be indeterminate in sign. Thus, the Second Law requirement of non-negative entropy production requires that their magnitude must be no greater than the explicit entropy production terms.

Although this conclusion is based on the MC implicit constitutive equation, it seems to us to have broader implications for other implicit constitutive equations that contain rate terms. A straightforward extension of our approach to generalized Maxwell viscoelastic fluid models will produce a combination of explicit entropy production terms and sign-indeterminate terms involving objective time derivatives of stress similar to (22). Moreover, as our analysis is based on the GAM principle, the conclusion applies immediately to single constituents.

## Data Availability

The original contributions presented in the study are included in the article, further inquiries can be directed to the corresponding author.
